# Emergency contraceptive utilization and associated factors among college students in Dire Dawa City, Eastern Ethiopia: A cross-sectional study

**DOI:** 10.18332/ejm/137655

**Published:** 2021-07-16

**Authors:** Legesse Abera, Alekaw Sema, Alemu Guta, Yalelet Belay

**Affiliations:** 1 Department of Midwifery, College of Medicine & Health Sciences, Dire-Dawa University, Dire Dawa, Ethiopia

**Keywords:** emergency contraceptives, utilization, college female students

## Abstract

**INTRODUCTION:**

Emergency contraception is a contraceptive method that can be used to prevent pregnancy after unprotected sexual intercourse. Higher education students fall under the sexually active age group and form a high-risk group for unintended pregnancy, because of limited utilization of emergency contraception. The aim of this study was to assess emergency contraceptive utilization and associated factors among college students in Dire Dawa City, Eastern Ethiopia.

**METHODS:**

An institutional-based cross-sectional study was conducted in May 2019. A total of 286 students using simple random sampling technique were included in the study. Data were collected by using self-administered questionnaires and entered into EpiData (Classic) Entry version 3.1 and analysed using SPSS version 24.0. Binary and multivariable logistic regression analysis were done to determine the association between the outcome and predictor variables.

**RESULTS:**

A total of 286 female students participated in the study giving a response rate of 100%. Eighty-six (86) participants had a history of sexual practice, and 60 (69.7%) had ever used emergency contraceptive. Having knowledge about emergency contraceptive (AOR=3.24; 95% CI: 1.32–7.98), age at first sexual intercourse ≥20 years (AOR=4.04; 95% CI: 1.72–9.52), history of previous pregnancy (AOR=3.12; 95% CI: 1.34–7.24) and previous use of regular contraceptives (AOR=5.01; 95% CI: 2.23–11.27) were found to be significant predictors of emergency contraceptive utilization.

**CONCLUSIONS:**

This study showed that emergency contraceptive utilization among female college students having unprotected sexual intercourse is still low. Level of knowledge, age at first sexual intercourse, previous use of regular contraceptives and history of pregnancy were major predictors of emergency contraceptive utilization. Therefore, focus on awareness creation activity and delaying sexual activity is recommended.

## INTRODUCTION

Emergency contraception is a contraceptive method that can be used to prevent pregnancy after unprotected sexual intercourse. Emergency contraceptive (EC) offers an important chance to prevent unintended pregnancy when a regular method fails, no method was used, or sex was forced^1^. For many years, the government and NGOs advocate to improve access to EC across the country. These efforts have been successful only in some urban areas, where EC is available. In other areas, despite the availability of highly effective methods of EC, many pregnancies that are unwanted and unplanned result in a high risk of morbidity and mortality^2,3^.

Globally, every day approximately 1000 women die from preventable causes related to pregnancy and childbirth. Adolescents face a higher risk of complications and death as a result of unplanned pregnancy than older women^4^. WHO estimates that at least 33% of all women seeking hospital care for complications related to abortions are less than 20 years of age. Currently, many young people especially college students engage in sexual activity before marriage often without using contraception resulting in unwanted pregnancies in many countries^5^. Worldwide 16 million adolescent girls aged 15–19 years give birth each year, roughly 11% of all births and 95% of these births occur in developing countries^6^.

The utilization of EC to prevent unwanted pregnancy among higher education students is low in Ethiopia, because of lack of knowledge. Without having knowledge, clients are unable to make an informed timely and appropriate contraceptive choice. On the other hand, the female’s negative attitude toward EC poses an equal challenge^7^. As the above literature revealed, there is existence of knowledge, attitude and practice gaps on EC among college students, so this study aims to assess EC utilization and identify the associated factors among college female students in Dire Dawa City, Eastern Ethiopia.

We found no similar study conducted in the study area, thus this study can serve to provide baseline data and help potential stakeholders to develop a strategy to improve emergency contraceptive method use in the study area.

## METHODS

### Study design and setting

An institution-based cross-sectional study design was conducted among 286 female students attending college in Dire Dawa city in May 2019. Dire Dawa (DD) is a known ancient city in Ethiopia, located about 500 km from Addis Ababa (capital city) in the Eastern part of Ethiopia. DD has an annual population growth rate of 2.9% and the region has a total population of 453000 (227000 males, 226000 females) and 94187 childbearing-age women. The total fertility rate for the region is 3.4 children/ woman^8^. The potential health service coverage of DD is 100% with two governmental hospitals, 15 health centres and 34 health posts. There are 4 private colleges in the city, namely Rift Valley, Lucy, Addis Ababa, and ART college^9^.

### Study participants

Female students were the study population in selected colleges, in the academic year 2019 and selected by simple random sampling. Critically ill students, night and weekend students were excluded from the study because as it was not possible to obtain data.

### Sampling method and sample size determination

Simple random sampling (SRS) technique was applied to select two colleges (Rift Valley and Lucy), after that number of students in each department were identified. The calculated sample size was proportionally allocated to each department in each year according to the number of students they had. Finally, the study subjects were selected within each department and year, by the SRS method. A sample size of 286 subjects was determined by using a single population proportion and population correction formula, considering p=53.5^10^ and adding a 10% nonresponse rate.

### Data collection tools, collection procedure and collectors

Data were collected by 5 nurses and 5 teachers in combination using structured self-administered questionnaires after orientation of the study participants, mainly on the aim of the study, on each part of the questionnaire, about consent, the right to participate or not, the right to withdraw at any time, and confidentiality issues. The study was carried out after getting permission from the ethical clearance committee of Dire-Dawa University. Data were collected after getting both oral and written informed consent from all study participants.

### Statistical analysis

Data were cleaned, edited, coded and entered into EpiData (Classic) Entry version 3.1, software and then exported to SPSS version 24.0 for analysis. Descriptive statistics such as frequencies, proportions and summary statistics were used to describe the study population in relation to relevant variables. Binary Logistic regression was used to assess the presence of association between predictors and outcome variable and variables having p<0.25 were a candidate for multivariable logistic regression. Odds ratio, p<0.05 with 95% CI were used to determine the significance, level of association between predictors and outcome variable.

### Measurements

The dependent variable was emergency contraceptive utilization and independent variables were sociodemographic factors, knowledge and attitude related variables and service-related factors.

### Data quality control

To ensure the quality of data, the questionnaire was translated to Amharic (local language) and back-translated to English. Pre-testing of the questionnaire was undertaken in 5% of female students in ART college before the actual data collection took place and corrections on the instrument were made accordingly. Training was given to all data collectors, supervisors, and facilitators. Close supervision was made by two teachers in each college. Data were checked for completeness, clarity and consistency by the facilitators and the principal investigator as soon as collected. Finally, data were entered through double data entry into EpiData (Classic) Entry version 3.1 software to minimize error during data entry.

## RESULTS

### Sociodemographic characteristics of respondents

A total of 286 female students participated in the study, making a response rate of 100%. The mean age of the respondents was 21.5 (SD: 2) years. With regard to religion, 109 (38.1%) of the respondents were Muslim followed by Orthodox Christian 102 (35.7%). The majority, 115 (40.2%), of participants were Oromo followed by Somali, 100 (35.0%), and most were unmarried/single, 229 (80.0%). Nearly half (45.5%) of respondent’s family average monthly income was less than 5000 Ethiopian Birrs (1000 Birrs about 23 US$), and for 30% it was 5000–10000 Ethiopian Birrs (Table 1).

**Table 1:** Sociodemographic characteristics of respondents on emergency contraception utilization and associated factors in private colleges of Dire Dawa city, Eastern Ethiopia, May 2019 (N=286)

*Variables*	*n*	*%*
**Age** (years)		
15–19	117	40.9
20–24	162	56.7
≥25	7	2.4
**Year of study**		
First	100	35.0
Second	91	31.8
Third	75	26.2
Fourth	20	7.0
**Field of study**		
Non-health sciences	141	49.3
Health sciences	145	50.7
**Marital status**		
Single	229	80.0
Married	51	17.8
Divorced	6	2.0
**Religion**		
Orthodox	102	35.7
Muslim	109	38.1
Protestant	60	21.0
Catholic	15	5.2
**Ethnicity**		
Oromo	115	40.2
Sumale	100	35.0
Amhara	43	15.0
Other^a^	28	9.8

a Other ethnic group: Adare, Tigre, Gurage or Hadiya.

### Sexual and reproductive health history (exposure)

**Figure 1:**
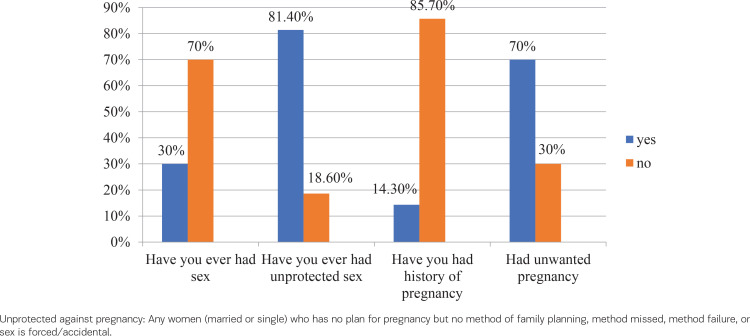
Sexual and reproductive health history of respondents on emergency contraceptive utilization and associated factors among female students, in private colleges of Dire Dawa city, Eastern Ethiopia, May 2019 (N=286)

Eighty-six (30.0%) participants were sexually active, of whom 60 (69.8%) were aged 15–19 years and 26 (30.2%) aged ≥20 years. Among participants having sexual experience, 70 (81.4%) had unprotected sex, 10 (14.3%) had been pregnant, and 7 (70%) had unintended pregnancies (Figure 1).

### Knowledge of emergency contraception

More than half (55.9%) of the study participants had heard about EC, but only 45 (28.1%) correctly identified time of administration of the EC, 25 (15.6%) the recommended doses, and 24 (15.0%) the recommended number of doses and the time interval between the doses. Fifty (31.3%) of the study subjects reported EC is less effective and only 25 (15.7%) of respondents reported that EC pills were greater than 95% effective. Overall, the knowledge summary index shows the majority, 200 (70.0%), of the respondents had poor knowledge and only 86 (30%) had good knowledge of EC (Table 2).

**Table 2:** Knowledge of female students on contraception, in private colleges of Dire Dawa city, Eastern Ethiopia, May 2019 (N=286)

*Knowledge assessment items/variables*	*n*	*%*
**Where do you think EC could be obtained** (n=160)		
Pharmacy/health facility	110	68.8
Any shop	48	31.2
**Which one of these drugs can be used for EC** (n=160)		
Pills and IUCD	98	29.5
Injectable	20	27.1
Implanol	42	10.2
**The recommended maximum time limit to take EC pills**		
Within 72 h after sex	25	28.3
Within 5 days after sex	123	12.7
Don’t know	12	22.9
**Recommended dose of EC pills** (n=160)		
1	90	15.1
2	60	16.3
3	5	10.5
Don’t know	5	58.1
**Recommended hours between the doses of EC pills** (n=160)		
12	49	14.8
24	45	13.5
72	50	15.1
Don’t know	16	56.6

### Attitude towards emergency contraception

**Figure 2:**
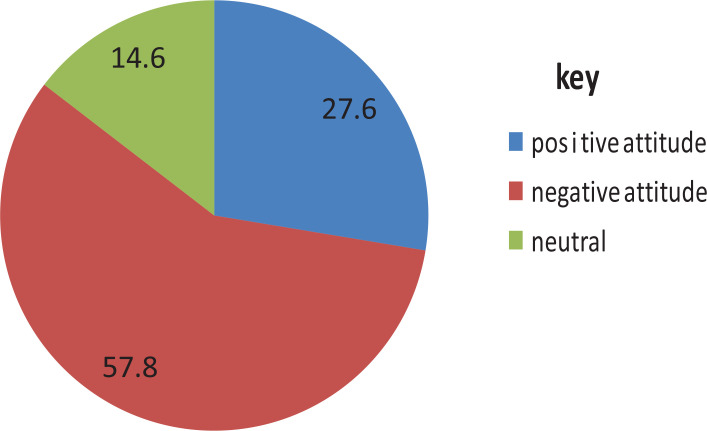
Showing percent of general (summary) attitude of respondents toward emergency contraceptive utilization and associated factors among female students, in private colleges of Dire Dawa city, Eastern Ethiopia, May 2019 (N=286)

Half, 144 (50.3%), of the respondents agreed that utilization of EC causes infertility in a woman. Concerning the overall level of female students’ attitudes, the majority 165 (57.8%) of the participants had an unfavourable/negative attitude and 79 (27.6%) had a favourable/positive attitude towards EC (Figure 2).

### Emergency contraceptive utilization

Among 86 participants who were sexually active, 70 (81.4%) had unprotected sex, of whom 60, 69.7% (95% CI: 65.9–72.3), had used an emergency contraceptive following unprotected sex. Friends were the major source of information for EC users. Among sexually active respondents who did not use EC, lack of knowledge about EC (20%), unavailability of EC (39%) and fear of being seen by others (10.3%), were the main reasons.

### Predictors of emergency contraception utilization

The multivariable logistic regression analysis showed that, respondents who had first sexual intercourse aged ≥20 years were 2 times more likely to use an emergency contraceptive compared to those who had their first sexual intercourse at a younger age (15–19 years), (AOR=2.04; 95% CI: 1.72– 6.5). Respondents who had a history of previous pregnancy were 3 times more likely to use an emergency contraceptive than those with no history of pregnancy (AOR=3.0; 95% CI: 2.3–9.02). Similarly, participants having good knowledge of emergency contraception were 2.2 times more likely to use an emergency contraceptive than those who were not knowledgeable about emergency contraception (AOR=2.24; 95% CI: 1.32–5.8). The result also showed that respondents who had experience of other forms of regular contraception utilization were 3 times more likely to use an emergency contraceptive than those who did not have exposure of other forms of regular contraception (AOR=3.01; 95% CI: 2.13–7.27) (Table 3).

**Table 3:** Predictors of emergency contraceptive utilization among female students, in private colleges of Dire Dawa city, Eastern Ethiopia, May 2019 (N=286)

*Variables*	*Used EC*		*Odds ratios*	
	*Yes n (%)*	*No n (%)*	*OR (95% CI)*	*AOR (95% CI)*
**Age** (years)				
≥20	40 (66.7)	130 (57.5)	1.89 (1.02–3.50)	2.12 (0.45–9.98)
15–19	20 (33.3)	96 (42.5)	1	1
**Year of study**				
Year II and above	46 (76.6)	153 (67.7)	2.15 (1.07–4.32)	0.60 (0.13–2.79)
Year I	14 (23.4)	73 (32.3)	1	1
**Field of study**				
Health sciences	23 (38.3)	130 (57.5)	1.69 (0.86–3.34)	0.52 (0.18–1.48)
Non-health sciences	37 (61.7)	96(42.5)	1	1
**Marital status**				
Ever married	15 (25.0)	42 (18.6)	5.26 (2.3–11.98)	2.90 (0.95–8.78)
Single	45 (75.0)	184 (81.4)	1	1
**Age at first sexual intercourse** (years)				
≥20	41 (68.3)	12 (22.6)	7.2 (3.71–14.18)	2.04 (1.72–6.5)*
15–19	19 (31.7)	41 (77.4)	1	1
**History of pregnancy**				
Yes	6 (10.0)	4 (40.0)	5.7 (2.95–10.84)	3.0 (2.3–9.02)*
No	54 (90.0)	6 (60.0)	1	1
**Ever use regular contraceptives**				
Yes	40 (66.7)	31 (25.8)	6.0 (3.12–11.52)	3.1 (2.13–7.27)*
No	20 (33.3)	89 (74.2)	1	1
**Knowledge on EC**				
Good	52 (86.7)	60 (19.0)	11.0 (5.45–22.53)	2.24 (1.32–5.8)*
Poor	8 (13.3)	256 (81.0)	1	1
**Attitude towards EC**				
Favourable	28 (46.7)	70 (22.2)	2.14 (1.11–4.13)	1.95 (0.80–4.75)
Unfavourable	32 (53.3)	246 (77.8)	1	1

AOR: adjusted odds ratio;

* Statistically significant association (p<0.05)

## DISCUSSION

Although emergency contraception (EC) is not recommended as a regular family planning method, it is a useful method after unprotected sexual intercourse to reduce the chance of unwanted pregnancies^11^.

The finding of this study revealed that, 69.7% (95% CI: 65.9–72.3) of the study participants having a history of sexual practice used EC following unprotected sex, which is higher than the results of the studies conducted in Dessie town, North East Ethiopia 51%^12^, Harar, Ethiopia 33%^13^, Wallo University, Ethiopia 30.9%^14^, Debre Markos University, Ethiopia 11.4%^15^, Jimma University, Ethiopia 22.2%^16^, Jimma University 41.9%^17^, Adama University, Ethiopia 34.8%^18^, Kampala University, Uganda (45.1%)^19^, and Princeton University, Kenya (8%)^20^. The possible reason for such variation could be time variation related to currently accelerated reproductive health promotion activities and youth friendly programs in the country and the increasing availability of EC in many government and non-government health institutions.

This study also showed that 30% and 27.6% of female students were found to have good knowledge and a positive attitude towards EC utilization, respectively. This finding is inconsistent with the studies conducted in Mizen Tepi, with values 24.1% and 46.8%, respectively^21^. The possible explanation for the differences observed could be related to differences in the sample size between the two studies. It might be also attributed to the differences in the provision of sexual and reproductive health education at schools and higher learning institutions, better practice of open and free discussion on sex and sexuality among female students varying from region to region. The result is similar to the finding of a study conducted in Uganda, where EC utilization was found to be higher among female students with a history of pregnancy and with knowledge of emergency contraception than their counterparts^19^. The lesson learned from a previous pregnancy and increasing awareness on how to prevent an unwanted pregnancy might be a possible explanation for the higher practice of emergency contraception among female students.

Participants that started their first sexual intercourse at age ≥20 years were found to be 2 times more likely to use an emergency contraceptive than those who started at an earlier age (≤19 years). This finding is similar to a study conducted in Adama University, Ethiopia^18^ and Harar, Ethiopia^13^. This could be due to better exposure to information or increased awareness about EC, maturity, and experiences of the consequences of unintended pregnancies held by these girls who started sex at an older age compared to those who had first sex at earlier ages.

Participants who had used regular contraception previously were 3 times more likely to use EC than those who had no previous experience. This finding is consistent with the studies done in Adama University^18^. This might be related to the experience of using different family planning services including EC by those who had the exposure than those who did not. Higher frequency of sex among female students who had used regular contraception could also be the other possible explanation for better utilization of EC.

### Limitations and strengths

Self-reported information could be subjected to reporting bias, and since the study touches on sensitive issues the possibility of underestimation cannot be excluded.

## CONCLUSIONS

Emergency contraception use among students who had unprotected sexual intercourse is still low. Participant’s level of knowledge, age at first sexual intercourse, previous use of regular contraceptives and history of pregnancy, were major predictors of EC utilization. Therefore, focus on awareness creation activity and delaying sexual activity is recommended.

## Data Availability

The data supporting this research is available from the authors on reasonable request.
